# Family caregivers’ contributions to self-care behaviors among heart failure patients in Oman

**DOI:** 10.1371/journal.pone.0319827

**Published:** 2025-03-14

**Authors:** Maryam Alharrasi, Huda Alnoumani, Amal Al-Ghassani, Anandhi Amirtharaj, Wafaa Bin Ali, Ibrahim Al-Zakwani, Laila Aldaken, Mahmood Al Hinai, Ahmad H. Abu Raddaha

**Affiliations:** 1 Adult Health and Critical Care Nursing Department, College of Nursing, Sultan Qaboos University, Muscat, Oman; 2 Community Health Nursing Department, Oman College of Health Sciences, Muscat, Oman; 3 Critical Care Nursing Department, College of Nursing, King Saud bin Abdulaziz University for Health Sciences, Jeddah, Saudi Arabia; 4 Pharmacology & Clinical Pharmacy, College of Medicine and Health Sciences, Sultan Qaboos University, Muscat, Oman; 5 Basic Nursing Department, Faculty of Nursing, Isra University, Amman, Jordan; 6 High Dependency Unit, Royal Oman Police Hospital, Muscat, Oman; 7 Pre-Clinical Affairs Department, College of Nursing, QU Health Sector, Qatar University, Doha, Qatar; BRAC Business School, BRAC University, BANGLADESH

## Abstract

**Background:**

Family caregiver’s role can be involving patients with heart failure (HF) in each behavior of self-care such as treatment adherence, and healthy eating, which will \ultimately lead to disease control. This study aimed to investigate family caregivers’ contributions to self-care behaviors among patients with heart failure in Oman.

**Methods:**

A descriptive cross-sectional design was used. A convenience sample of 136 family caregivers of patients with HF has completed the family caregivers’ demographics characteristics sheet and the Caregiver Contribution to Self-Care of HF Index2 (CC-SCHFI 2).

**Results:**

Caregivers demonstrated low levels of contribution to patients’ self-care. The mean and (standard deviation) of caregivers’ contribution to maintenance tasks, patients’ ability to perceive symptoms, and to patients’ ability to manage self-care tasks scored 64.12 (SD = 15.70), 66.78 (SD = 14.72).and 52.26 (SD = 15.98) respectively. Education, exercise, and quality of social support were found to have a statistically significant association with caregivers’ contribution to self-care maintenance at a p–value of 0.004, 0.004, 0.004 respectively. While gender, education, marital status, exercise, and quality of social support had statistically significant association with caregivers’ contribution to self-care perception at a p-value of 0.003, 0.002, 0.006, < 0.01, and 0.004 respectively. Moreover, gender, education, marital status, exercise, and health compared to 1 year ago had significant association with caregivers’ contribution to self-care management at a p-value of 0.009, 0.006, 0.005, <  0.01, 0.007 respectively.

**Conclusion:**

Giving attention and support to caregivers can indirectly impact the self-care behaviors of patients with HF and consequently enhance patients’ outcomes.

## Introduction

Cardiovascular related illnesses are among leading diseases of high mortality globally [1]. Heart failure (HF) is one of the most common reasons for hospitalization among those cardiac diseases due to its complexity and its progressive incidence [2]. It is estimated that 64.3 million people are living with HF worldwide [3]. Further, this number is expected to increase with an ageing population. There are several psychological and physical symptoms that HF patients experience, which may affect their general quality of life. These symptoms include fear, isolation, depression, chest pain, malaise, and shortness of breath [4]. Different research concluded that self-care activities reduce readmission to hospitals significantly, reduce care cost, and eventually mortality rate. Further, patients with HF usually find it challenging to perform self-care activities related to disease complications [5]. Therefore, the availability of good family support for self-care is required.

Self-care is linked with improving patients’ self-care management in chronic illnesses [5]. Families and caregivers can be involved in HF patients’ self-care such as treatment adherence, and healthy eating, which will lead ultimately to disease control [6]. Family contribution can also be made by doing the action for the HF patient if the patient is unable to do so [6]. Family is considered an important source of social support, contributing directly to self-care. There is an increased amount of literature that supports the association between family and social support with HF patients’ positive health outcomes [7]. Family can influence the HF patient’s consistency of their self-care behaviors. Positive family contribution results in positive HF patient outcomes, such as maintaining healthy behaviors and more compliance with healthy behaviors [7]. On the other hand, literature indicated that some family factors such as inappropriate conflict resolutions and problem solving, aggressiveness, mistrust and poor family relationships lead to poor chronic disease patients’ outcomes [7]. Also, factors such as living alone, and isolation are shown to be associated with higher mortality and morbidity rates. HF patients who lack family support like those who are not married, or live alone report having signs of depression and showed poor quality of self-care [8]. Patients with heart failure living without family are at a higher risk to exhibit low or no self-care [9].

A recent Scientific Statement by the American Heart Association highlighted the necessity of systematically assessing the impact of heart failure caregiving in observational studies [10]. Professionals and informal caregivers are encouraged to consider concepts related to service integration between institutions and family. Poor coordination between family caregivers and institutions negatively affects the quality of care provided to patients [11]. Considering the factors that impact family contribution in care, it is essential to identify ways to create effective long-term linkages among family members as caregivers, social systems, and formal care institutions. It was found that the following strategies are essential throughout the continuum of care among people’s chronic diseases and their informal caregivers: establishing a trusting relationship with caregivers and professionals, having professional knowledge and commitment, and offering varied services and care adapted to patients’ needs [12]. Family care givers burden was linked directly with low quality of life [13]. Factors such as family burden, insufficient care provided by family, financial burden, poor communication, and collaboration between professionals and family caregivers, and lack of services from the public health care system are associated with poor health outcomes for HF patients and their immediate care providers, as well as increased hospitalization [14].

Families as caregivers often feel that they are not prepared to provide care or have inadequate knowledge and skills of care delivery [14]. Reinhard et al. [15] considered families as caregivers as “secondary patients”. On the other hand, families who play the role of caregivers to HF patients need to be updated about disease processes and management to provide better support to those patients. Nurses and health care providers have the responsibility to help in improving family knowledge to play the role of support, education, and counseling [12]. Families as caregivers may experience a disrupted emotional, cognitive, and functional status due to increased burden from prolonged care provided to patients suffering from chronic diseases. This status can be manifested by feelings of fear, isolation, and insomnia [14]. Also, it is highly important for caregivers to have trusting relationships with professionals, to receive professional guidance and knowledge, and to receive a variety of services and care adapted to their patients’ needs. This will help the family caregivers to be competent and confident when providing care, which would ultimately reduce caregiver’s feeling of distress and enhance their confidence level. It is shown that caregivers’ burden is reduced significantly when provided with social support groups and education. As well, it was evidenced that comprehensive counseling sessions helped in reducing signs of depression among family members caring for a spouse with a chronic condition [6].

Although many studies have highlighted the important role of family support in heart failure patients’ self-care, there is a need for more research to explore this concept in diverse cultural contexts, including Oman, where no studies have specifically addressed this topic [10]. In Oman, there are no formal policies supporting family caregivers, and the healthcare infrastructure offers limited options for formal caregiving, such as nursing care facilities or elderly day care centers. Cultural norms place the responsibility of caregiving on family members, often with limited access to professional caregivers or support systems. Additionally, many patients lack formal education, and the healthcare system does not provide structured follow-up after hospital discharge, further increasing the burden on family caregivers. This study aimed to investigate family caregiver contributions to self-care of heart failure patients in Oman.

## Methodology

### Study design and sample

This was a cross-sectional study. The convenience-sampling technique was used to select family caregivers of HF patients from the outpatient clinics of major hospitals in the five largest cities in the country. Caregivers were included if they were adults aged 18 years and above, caring for a patient diagnosed with HF, able to communicate their experiences, English or Arabic speakers, and willing to participate in the study. Participants were excluded from participation if they had a cognitive impairment, major psychiatric illness (e.g., depression), or concurrent terminal illness (e.g., terminal cancer). The caregivers’ sample size was estimated using a rule of thumb of 25 participants per covariate for regression analysis [16]. In this study we examined 9 covariates so the estimated sample size for this study is 225 subjects. However, the total number of participants who responded was 136.

### Study procedure

Ethical approval of the study was granted from the Research and Ethics Committee of the Ministry of Health in Oman. Data were collected by research assistants (RAs) trained in how to screen and consent participants for the study and collect the data. After granting ethical permission to conduct the study, the RAs contacted the outpatient clinic facilities and explained the study to the nurse managers. A follow-up list of patients with HF was obtained from each site. Then RAs screened patients with HF to identify eligible family caregivers. Caregivers were selected either on the day of the next clinic follow-up of their patients or through a phone call.

An elaborate description of the study’s purpose, procedure, and how long it would take was given to those who were approached. When they indicated their willingness to take part, those individuals were required to consent by putting their signature on a form that had the consent agreement printed on it. Participants’ information confidentiality and privacy were maintained throughout the study period. Participants were assured that no identified data will be published and that their participation is voluntary and can withdraw from the study at any time without any harm. Participants were then asked to complete self-report questionnaires that took 20 minutes to complete.

## Measures

A questionnaire was developed by the researchers and consists of the following sections. The first section is demographics questions, where the researchers ask the subjects questions related to their gender, age, education, and marital status, and health behaviors. In the second section, the Caregiver Contribution to Self-Care of HF Index2 (CC-SCHFI 2) was used. This standardized self-report tool was created by Vellone et al. in 2013 — an adaptation from the HF self-care tool (Version 6.2) — with the primary objective of evaluating the effects of caregiver participation in HF patient self-care [17]. The CC-SCHFI 2 comprises 29 items rated on a 5-point Likert scale (ranging from “never” to “always”), with total scores ranging from 29 to 145, where higher scores indicate better self-care. It consists of three subscales: Caregiver Contribution to Self-Care Maintenance (items 1–10), Caregiver Contribution to Self-Care Perception (items 11–21), and Caregiver Contribution to Self-Care Management (items 22–29). A score of 70 or above is considered favorable for contribution to self-care. In Vellone et al.‘s study, the overall reliability of the tool, assessed using Cronbach’s alpha, was 0.94, with subscale reliability ranging from 0.7 to 0.85 [12].

To ensure linguistic and conceptual equivalence, the items were translated from English to Arabic and then back-translated by an English-fluent expert, following the WHO guidelines. Permission to use and/or translate the tool was obtained from its original developers. The tool has been proved to be valid in countries with similar cultural context [20]. In this study, reliability was measured through internal consistency, Cronbach’s alpha for the maintenance scale was .69, for the Perception scale .77, and .84 for the management scale.

### Data analysis

A comprehensive check for missing data and evaluation whether our data adhered to normal distribution, uniform variance consistency and the homogeneity assumption was performed before the data analysis. We presented descriptive statistics such as means, standard deviations and percentages of caregiver characteristics of HF patients.

To investigate relationships between various variables enlisted in Table 1 with caregiver’s contribution subscales as depicted in Table 2, bivariate correlation analysis was used. We looked closely at the Pearson correlation coefficients produced, through a two-tailed test of significance. This was done to decide on the variables that should be used in building regression models. The significance level was set at p-values less than .05.

**Table 1:** Caregivers’ sociodemographic and health characteristics (N =  136).

Characteristics		n (%)
**Gender**	Male	79(58.1)
	Female	57(41.9)
**Age** (Years), mean (SD)	40.14 ± 14.424	
**Education**	Elementary	28 (20.6)
	Middle school	50 (36.8)
	Professional school	22 (16.2)
	High school	1(0.7)
	University degree	35 (25.7)
**Marital Status**	Married	101(74.3)
	Single	28(20.6)
	Widowed	1(0.7)
	Divorced	6(4.4)
**Type of Kinship**	Spouse	50(36.8)
	Child	60(44.1)
	Friend	4(2.9)
	Nephew/niece	4(2.9)
	Brother/sister	10(7.4)
	Other relative	8(5.9)
**Employment**	Employed	67(49.3)
	Unable to work or on sick leave	36(26.5)
	Medical retirement	1(.7)
	Not Employed	12(8.8)
	Home maker	1(.7)
	Retired	19(14.0)
**Financial Status**	Do not have Enough	108(79.4)
	Have Enough	28(20.6)
**History of Smoking**	Current smoker	9(6.6)
	Former smoker (quit more than a year)	1(0.7)
	Recently quit smoking (less than a year)	3(2.2)
	Never smoked	123(90.4)
**Alcohol Drinking**	Never had Alcohol	134(98.5)
	8-14 per week	2(1.5)
**Exercise in the Last Week**	Didn’t Exercise	22(16.2)
	30m-1h	33(24.3)
	More than 3 hours	31(22.8)
	Less than 30 m	50(36.8)
**Have Someone to Confine in**	Yes	133(97.8)
	No	3(2.2)
**Quality of Social Support**	Bad	2(1.5)
	Acceptable	64(47.1)
	Good	24(17.6)
	Very Good	46(33.8)
**Self-rated Health**	Excellent	57(41.9)
	Very Good	10(7.4)
	Good	45(33.1)
	Poor	24(17.6)
**Current Health Compared to 1 Year Ago**	Much better now than 1 year ago	36(26.5)
	About the same as 1 year ago	25(18.4)
	Much worse now than 1 year ago	70(51.5)
	Somewhat better now than 1 year ago	5(3.7)

**Table 2:** Caregivers’ contribution.

	Minimum	Maximum	Mean	Std. Deviation
Maintenance	30	100	64.12	15.698
Symptom Perception	27	98	66.78	14.724
Management	20	80	52.26	15.983

To understand the association among the dependent and independent variables linear regression was used. We included significant variables in the regression model, as well as additional variables known to have a relationship based on previous research. Variables included in the regression model were age, gender, education, marital status, employment, exercise, type of kinship, quality of Social Support, self-rated health and health compared to 1 year ago. All analyses were performed using IBM Statistical Package for the Social Sciences (IBM SPSS version 29).

## Results

### Sample characteristics

136 eligible caregivers of Heart failure patients completed the questionnaire. The mean age of the caregivers was 40.14 ± 14.42 years. Majority of caregivers were male (58.1%). Of the 136 caregivers, 101 (74.3%) were married with 36.8% having middle school level of education, 49.3% are employed and 79.4% indicated having adequate income. This sample was relatively healthy in terms of lifestyle habits, where 90.4% were non-smokers, and 98.5% do not drink alcohol. However, in terms of activities nearly 90% of the participants either didn’t exercise or exercise less than 30 minutes per week. More than 80% of the caregivers rated their health as good, very good or excellent. Compared to 1 year ago, most of the caregivers indicated that their health has worse (50.5%). Table 1 displays the sociodemographic and health characteristics of the caregivers.

### Caregivers’ contribution to self-care of patients with HF

In general, all the averages of caregivers’ contribution to self-acre were less than a score of 70. The lowest score in terms of contribution to self-care maintenance was 30, while the highest score was 100. On average, caregivers scored around 64.12 (SD = 15.70) in their contribution to maintenance tasks. Among the 136 caregivers, the lowest score recorded was 27, while the highest was 98 for the contribution to Symptom Perception. On average, caregivers scored approximately 66.78 (SD = 14.72) in their contribution to patients’ ability to perceive symptoms. Caregivers’ contribution to self-care management scores ranged from 20 to 80. The average caregiver score on self-care management was 52.26 (SD = 15.98). Overall, the scores of caregivers’ contribution falls below 70 across maintenance, symptom perception, and management subscales. Table 2 shows scores of each subscale.

Frequencies and percentages of caregivers’ contribution items is presented Table 3. Several items showed high levels of caregivers’ contribution. Majority of caregivers reported “Always” or “Very Quickly” contributing to behaviours, i.e., trying to avoid getting sick (78.70%), taking prescribed medications regularly (79.40%), asking healthcare providers about medications (67.60%), monitoring weight daily (64.00%), being attentive to changes in feeling (50.00%), noticing fatigue in the patient (51.50%), asking about the patient’s well-being (51.50%), watching for symptoms intensively (57.40%), and examining ankles for swelling (44.90%). Some caregivers reported less engaging “Never” or “Less than sometimes”, in important actions such as ordering low-salt foods when eating out, getting the flu shot annually, asking for low-salt food when visiting family and friends and using a system to remember medication.

**Table 3:** Responses to the items.

	Item	Response	Frequency	Percent
1	Try to avoid getting sick (e.g., wash your hands)	Never	3	2.20
		Less than sometimes	1	0.70
		Sometimes	25	18.40
		Always	107	78.70
2	Do some exercise (e.g., do light walks, use the stairs instead of the elevator)?	Never	14	10.30
		Sometimes	74	54.40
		Always	48	35.30
3	Eat low-salt food	Never	12	8.80
		Sometimes	87	64.00
		Always	37	27.20
4	See your healthcare provider for regular check-ups	Never	9	6.60
		Sometimes	56	41.20
		Always	71	52.20
5	Take prescribed medications regularly and without forgetting any dose?	Never	4	2.90
		Sometimes	24	17.60
		Always	108	79.40
6	Order low-salt foods when you eat in restaurants?	Never	37	27.20
		Sometimes	69	50.70
		Always	30	22.10
7	Make sure you get the flu shot every year?	Never	47	34.60
		Sometimes	55	40.40
		Always	34	25.00
8	Ask for low-salt food when visiting your family and friends?	Never	48	35.30
		Sometimes	72	52.90
		Always	16	11.80
9	Use a system to help you remember to take medications?	Less than sometimes	46	33.80
		Sometimes	49	36.00
		Always	41	30.10
10	Ask your healthcare provider about medications?	Less than sometimes	5	3.70
		Sometimes	39	28.70
		Always	92	67.60
11	Monitor weight on a daily basis?	Less than sometimes	30	22.10
		Sometimes	87	64.00
		Always	19	14.00
12	Attentive to changes in feeling?	Less than sometimes	12	8.80
		Sometimes	56	41.20
		Always	68	50.00
13	Know the side effects of medications?	Less than sometimes	10	7.40
		Sometimes	59	43.40
		Always	67	49.30
14	Notice if the person feels more tired than usual when doing normal activities?	Less than sometimes	6	4.40
		Sometimes	60	44.10
		Always	70	51.50
15	Ask the caretaker/patient how he is?	Less than sometimes	6	4.40
		Sometimes	60	44.10
		Always	70	51.50
16	Watch for symptoms intensively?	Less than sometimes	2	1.50
		Sometimes	56	41.20
		Always	78	57.40
17	Examine ankles for swelling?	Less than sometimes	25	18.40
		Sometimes	50	36.80
		Always	61	44.90
18	Check for shortness of breath with activities such as bathing and dressing?	Less than sometimes	20	14.70
		Sometimes	62	45.60
		Always	54	39.70
19	Keep a record of symptoms?	Less than sometimes	52	38.20
		Sometimes	57	41.90
		Always	27	19.90
20	How quickly do you spot your symptoms?	I did not recognize the symptom	1	0.70
		Not Quickly	8	5.90
		Not so Quickly	11	8.10
		Somewhat Quickly	31	22.80
		Somewhat Quickly	76	55.90
		Very Quickly	9	6.60
21	How quickly did you know that the symptom was due to heart failure?	I did not recognize the symptom	2	1.50
		Not Quickly	13	9.60
		Not so Quickly	25	18.40
		Somewhat Quickly	37	27.20
		Somewhat Quickly	53	39.00
		Very Quickly	6	4.40
22	Further limit the salt he/she eats that day?	Not Likely	52	38.2
		Somewhat Likely	57	41.9
		Very Likely	27	19.9
23	Reduce fluid intake?	Not Likely	4	2.90
		Somewhat Likely	53	39.00
		Very Likely	79	58.10
24	Take a medicine?	Not Likely	20	14.70
		Somewhat Likely	62	45.60
		Very Likely	54	39.70
25	Call the health care provider for guidance?	Not Likely	25	18.40
		Somewhat Likely	50	36.80
		Very Likely	61	44.90
26	Ask a family member or friend for advice?	Not Likely	52	38.20
		Somewhat Likely	57	41.90
		Very Likely	27	19.90
27	Try to figure out why he/she has symptoms?	Not Likely	4	2.90
		Somewhat Likely	53	39.00
		Very Likely	79	58.10
28	Suggest that he/she limit activity until he/she feels better?	Not Likely	20	14.70
		Somewhat Likely	62	45.60
		Very Likely	54	39.70
29	Did the treatment you used make him/her feel better?	I did not do anything	6	4.40
		Not Sure	23	16.90
		Somewhat Sure	45	33.10
		Somewhat Sure	1	0.70
		Very Sure	61	44.90

### Associated factors with caregiver contribution to self-care maintenance

Among the factors analyzed for their association with caregiver contribution to self-care maintenance, only three were found to have a statistically significant association. Specifically, education (P = .004), exercise (P = .004), and quality of social support (P = .004) showed significant association with self-care maintenance. That is, caregivers who have higher educational level, have higher exercise rate, and have better quality of social support, have also higher contribution to self-care maintenance. In contrast, factors such as age, gender, marital status, employment, type of kinship, self-rated health, and health compared to one year ago did not demonstrate a significant association with self-care maintenance.

### Associated factors with caregiver contribution to self-care perception

Several factors showed significant associations. Gender, education, marital status, exercise, and quality of social support had statistically significant association with self-care perception at a p-value of 0.003, 0.002, 0.006, < 0.01, and 0.004, respectively. That is, participants who were female, have higher educational level, married, have higher excise rate, have better quality of social support have also better contribution to self-care perception. On the other hand, age, employment, type of kinship, self-rated health, and health compared to one year ago did not have a significant relationship with self-care perception.

### Associated factors with caregiver contribution to self-care management

**Fig 1:**
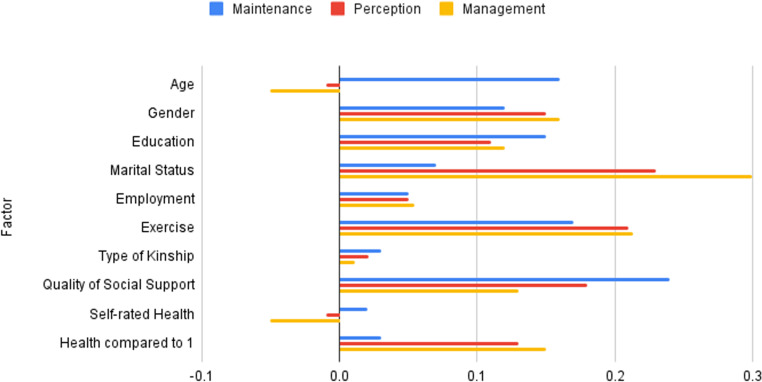
Factors related to care givers contribution to self-care.

Gender (p =  0.009), education (p =  0.006), marital status (p =  0.005), exercise (p <  0.01), and health compared to 1 year ago (p =  0.007) were found to have a significant association with contribution to self-care management. That is, participants who were female, have higher educational level, married, have higher excise rate, and have better health compared to 1 year ago, have also better contribution to self-care perception. The remaining factors, age, employment, type of kinship, quality of social support, and self-rated health, were not found to be significant contributors to self-care management. Table 4 and Fig 1 shows the details of associated factors with caregiver contribution.

**Table 4:** Factors influencing family caregiver contribution to heart failure selfcare (Model R-square).

	Contribution to Self-Care Maintenance					Contribution to Self-Care Perception					Contribution to Self-Care Management				
				95% CI					95% CI					95% CI	
Factor	β	t	P	Lo.	Up.	β	t	P	Lo.	Up.	β	t	P	Lo.	Up.
Age	.16	1.4	1.65	−.106	.614	−.01	−2	.900	−.18	.148	−.05	−.5	.640	−.42	.26
Gender: Female	.12	1.7	.083	−.70	11.1	.15	3.1	**.003**	1.5	6.83	.16	2.7	**.009**	1.9	13.0
Education: have a university degree	.15	2.9	**.004**	1.00	5.13	.11	3.1	**.002**	.53	2.39	.12	2.8	**.006**	.80	4.7
Marital Status: Married	.07	.62	.530	−5.08	9.80	.23	2.8	**.006**	1.4	8.1	.30	2.9	**.005**	3.0	17.0
Employment: Retired	.05	1.2	.230	−.83	3.30	.05	1.5	.14	−.24	1.62	.054	1.32	.200	−.64	3.22
Exercise: Exercise for 1-3 h a week	.17	3.0	**.004**	1.21	6.1	.21	5.0	**<0.001**	1.7	3.90	.213	4.2	**<0.001**	2.6	7.12
Type of Kinship: Child	.03	.70	.490	−1.31	2.73	.021	.700	.490	−.59	1.23	.011	.30	.770	−1.61	2.2
Quality of Social Support: Very good	.24	2.9	.**004**	1.61	8.23	.18	3.0	**.004**	.76	3.73	.13	1.8	.070	−.24	6.0
Self-rated Health: Fair	.02	.38	.700	−3.11	4.6	−.01	−.30	.800	−2.0	1.5	−.05	−.92	.360	−5.3	1.92
Health compared to 1 year ago: Somehow worse than 1 year ago	.03	.40	.680	−2.50	3.80	.13	3.0	**.003**	.730	3.6	.15	2.7	**.007**	1.10	7.00

CI, Confidence Interval, *  < .01, ** < .001, significance (P) was set at < .05. Reference Groups: Married, Do Not Drink Alcohol, Do Exercise, Poor General Health, Much worse now than 1 year ago, Class 1, Poor Quality of Social Support. NYHA: New York Heart Association.

## Discussion

This study aimed to investigate family caregiver contributions to self-care of heart failure patients in Oman. Caregivers in this study demonstrated suboptimal levels of contribution across various aspects of self-care of HF patients. Educational level, exercise rate, and quality of social support emerged as significant predictors of caregiver contributions to self-care maintenance. Similarly, gender, marital status, and perceived health compared to one year ago in addition to educational level, exercise rate were significant predictors of caregiver contributions to patient’s self-care perception and management. This might be due to the majority of caregivers in this study were male, married, and have completed middle school.

Our finding of low levels of caregivers’ contribution to self-care maintenance, perception, and management, was similar to other studies conducted in HF patients in other countries [18,19], but was unlike others who found self-care to be adequate [19,20]. For a variety of reasons, caregivers may not contribute as much as they should to their patients’ self-care practices. First off, there’s a chance that the caregivers in this sample don’t know much about managing heart failure or the value of practicing self-care. Caregivers might not fully comprehend their role in supporting patients’ self-care requirements or how to properly enable self-care activities if they lack the necessary education and awareness. Care provider education has been demonstrated to enhance self-care habits and lower hospital readmission rates in a recent randomized control research [21]. Higher caregiver confidence was associated with higher caregiver preparation scores, and higher caregiver confidence was associated with higher contributions to the maintenance and management of self-care [12]. Second, there’s a chance that the caregivers in this study encountered difficulties getting the informative materials, medical supplies, pharmaceuticals, and other resources they needed to help patients practice self-care.

We collected data from all over Oman and some geographical areas have a smaller number of health care facilities which may have led to limited access to care. Financial constraints, or geographic barriers may limit caregivers’ ability to access essential resources for patient care [14]. Thirdly, providing care demands a lot of time and energy, which leaves caregivers with little time to support patients’ practices of self-care. The majority of the caregivers in this study were married, young, and had jobs. Prioritizing patient self-care can be difficult for caregivers when juggling caregiving tasks with employment, household duties, and personal commitments [22–24]. Fourthly, providing care can be emotionally and physically taxing, which can wear out and burn out caregivers. The majority of caregivers in this study reported that compared to a year ago, their health had significantly deteriorated. Caregivers may become physically and emotionally worn out from managing the stress and emotional strain of providing care, making it difficult for them to continuously encourage patients’ self-care [25]. Fifth, family, friends, or medical professionals may not provide caregivers with enough support to help them fulfill their job as caretakers. In the absence of a robust support system, caregivers may experience feelings of isolation and be less equipped to handle the responsibilities of caregiving, such as encouraging patients to engage in self-care activities [10,18].

Like other studies educational level has positive association with increased contribution to self-care maintenance in this study. This may be because individuals with higher education levels have better health literacy, have better preparation, understanding complex aspects of heart failure management and supporting patients’ self-care effectively [12]. Critical thinking abilities are developed through education, which enables caregivers to evaluate tactics, spot obstacles, and improve self-care management. Additionally, access to healthcare services, information, and support networks is made possible by higher levels of education, which helps caregivers support patients in maintaining their health. However, in the current study most of the participants were found to have less than high school education which may have contributed to the suboptimal caregiver contribution to self-care maintenance. Of note in spite of the theoretical positive link between education and caregiver contribution to self-care, there are studies that found no significant relationship between the two [26].

The study’s results add to the body of evidence that underscores how vital physical activity is in predicting caregiver support to heart failure patients’ self-care. For individuals who have already demonstrated commitment to their care regimens, it can be easier for them when surrounded by caregivers who value regular exercise as part of healthy living. A caregiver’s behavior is an outcome of their personal health consciousness level, which can also reflect other supportive efforts towards care provision (e.g., psychological motivation or financial contribution). Moreover, positive effects from caregiver involvement in exercise create an inspiring environment that fosters long-term self-care behaviours contributing further not only based on benefits but more importantly from intrinsic rewards established by those actions themselves and perceived appreciation by the care recipient.

Quality of social support was found to play a crucial role in shaping caregiver contributions to self-care maintenance and perception for heart failure patients, like other studies [12,21]. Caregivers who feel supported and are more effective in helping patients in their self-care routines [10,21,27]. Furthermore, healthy social support can alleviate caregiver stress, leading to more consistent and positive engagement in patient care [28,29].

The finding that gender significantly predicts caregiver contributions to heart failure patient self-care underlines important dynamics in caregiving roles. It suggests that male and female caregivers may differ in their approaches, effectiveness, or willingness to engage in patient self-care activities [30]. Like other studies, we found higher scores of contributions by female caregivers compared to male scores [20].

We also found that marital status is a significant predictor of caregiver contributions to patient self-care which suggests that married caregivers may provide more effective support. This might be due to the presence of a stable partner, which can enhance emotional and practical mutuality [31]. This result is similar with Karimi et al.’s and Cohen et al.’s study [20,32]. However, it contrasts with Bidwell et al.’s study [33], where despite most caregivers being married, they reported low contributions to self-care.

Married caregivers might also benefit from shared responsibilities, reducing stress and burnout, thereby improving their ability to assist with self-care. Conversely, single caregivers may face greater challenges balancing caregiving with other obligations [34].

The finding that perceived health significantly predicts caregiver contributions to patient self-care emphasizes how caregivers’ own health perceptions affect their effectiveness in caregiving. Caregivers who perceive having a good health are likely more competent of providing consistent and quality support. Caregivers’ physical and psychological health impact their contributions to patient self-care, making it necessary to provide supportive interventions [35].

### Limitations

This study examined certain factors, leaving our understanding of the caregiver’s influence on self-care of patient with HF patients still limited. For example, inadequacies within the healthcare system, such as limited access to healthcare services, long wait times, or fragmented care, may hinder caregivers’ ability to successfully support patients’ self-care behaviors.

The descriptive research design used in this study describes the existing behaviour but fails to identify any relationship that isolates the cause and effect in the research. This design can be justifiably mentioned as providing the iceberg view of the problem for instances the caregiver role and deficiencies in care of the HF patient but does not identify the cause for the deficiencies in caring for the patient. In addition, a self- report questionnaire used for data collection to identify the involvement of the caregiver and its impact among the HF patients relies on the memory of the caregiver which may affect the accuracy. The tool which measured the three dimensions of self-care perception, management and maintenance raised certain specific information which does not guarantee accuracy based on one point in time data collection. The study’s low response rate (136 out of 225) limits generalizability, as the use of a convenience sample may have introduced nonresponse bias, affecting sample representativeness, however, analyses conducted on the study results indicate that the study has adequate statistical power to make inferences.

Although the CC-SCHFI2 was previously validated in a nearby country with similar cultural and linguistic characteristics, this study represents its first use in Oman. While validity testing specific to the Omani population was not conducted, the tool demonstrated high reliability in this study. Future research should aim to validate the instrument further within the Omani context to enhance its cultural sensitivity. Quantitively examining caregiver contribution to self-care limited our understanding of the suboptimal contribution level in self-care in this study. Using a qualitative approach might help in shading lights and giving the opportunity of caregivers to share their personal experiences in contributing to self-care for patients with HF. However, these findings can be further used for planning and executing further training sessions or proposing future studies based on the outcome.

### Implications

The implications of the study can be specifically enumerated in areas of clinical practice. To identify and study the impact of sub optimal knowledge affecting the quality of life among the HF patients. The outcomes can be measured linking the discharge planner and community nurse to identify the benefits of a customized teaching learning session. Discharge summary pamphlets regarding self- care contributions specifically focusing on the maintenance and management of HF patients can be provided with evaluations at timed interval of every OPD visit can be planned.

The medical cost cannot be measurably reduced, and steps must be taken to identify the reason for frequent which is due to lack of knowledge and confidence in caring for these patients. Reducing the chances of frequent hospitalization will reduce the financial burden, resource management and increase in the life expectancy of the patients. This study also emphasizes on the need for targeted support systems that consider marital status and access of resources to optimize caregiver effectiveness in patient self-care.

We recommend that educational sessions targeted toward involvement of caregivers in HF self-care can be beneficial to increase contribution of caregivers and influence HF patients’ health. Further research is warranted to explore other factors that are unique to the Omani culture.

## Conclusion

This study provides empirical evidence on caregivers’ contribution to self-care behaviors of HF patients. Caregivers in this study demonstrated suboptimal levels of contribution across various aspects of self-care of HF patients. Factors that can impact the contribution of caregivers in HF patients’ self-care behaviors need to be assessed and considered when implementing HF management programs unique to the Omani culture. Educational level, exercise rate, and quality of social support emerged as significant predictors of caregiver contributions to self-care maintenance. Gender, marital status, and perceived health compared to one year ago in addition to educational level, exercise rate were significant predictors of caregiver contributions to patient’s self-care perception and management. The family caregiver role is highly important in the management of HF and more attention should be given to support caregivers to improve HF patients’ health outcomes, namely health control and hospital readmission. Future studies may explore stage-specific caregiving experiences for greater depth in terms if these aspects.

## Supporting information

S1 File Data set file. (XLSX)

## References

[1] Roth GA, Mensah GA, Johnson CO, Addolorato G, Ammirati E, Baddour LM, et al. Global burden of cardiovascular diseases and risk factors, 1990-2019: update from the GBD 2019 study. J Am Coll Cardiol. 2020;76(25):2982–3021. doi: 10.1016/j.jacc.2020.11.010 33309175 PMC7755038

[2] Darvishzadehdaledari S, Harrison A, Gholami F, Azadnia A. Examining the effectiveness of home-based cardiac rehabilitation programs for heart failure patients with reduced ejection fraction: a critical review. BMC Cardiovasc Disord. 2023;23(1):593. doi: 10.1186/s12872-023-03640-x 38053086 PMC10696730

[3] Savarese G, Becher PM, Lund LH, Seferovic P, Rosano GMC, Coats AJS. Global burden of heart failure: a comprehensive and updated review of epidemiology. Cardiovasc Res. 2023;118(17):3272–87. doi: 10.1093/cvr/cvac013 35150240

[4] Podvorica E, Bekteshi T, Oruqi M, Kalo I. Education of the patients living with heart disease. Mater Sociomed. 2021;33(1):10–5. doi: 10.5455/msm.2021.33.10-15 34012343 PMC8116090

[5] Kolasa J, Lisiak M, Grabowski M, Jankowska EA, Lelonek M, Nessler J, et al. Factors associated with heart failure knowledge and adherence to self-care behaviors in hospitalized patients with acute decompensated heart failure based on data from “the weak heart” educational program. Patient Prefer Adherence. 2021;15:1289–300. doi: 10.2147/PPA.S297665 34163146 PMC8214567

[6] Hodson AR, Peacock S, Holtslander L. Family caregiving for persons with advanced heart failure: An integrative review. Palliat Support Care. 2019;17(6):720–34. doi: 10.1017/S1478951519000245 31134868

[7] Nicholas Dionne-Odom J, Hooker SA, Bekelman D, Ejem D, McGhan G, Kitko L, et al. Family caregiving for persons with heart failure at the intersection of heart failure and palliative care: a state-of-the-science review. Heart Fail Rev. 2017;22(5):543–57. doi: 10.1007/s10741-017-9597-4 28160116 PMC5544594

[8] Yodmai K, Somrongthong R, Nanthamongkolchai S, Suksatan W. Effects of the older family network program on improving quality of life among older adults in Thailand. J Multidiscip Healthc. 2021;14:1373–83. doi: 10.2147/JMDH.S315775 34135595 PMC8197577

[9] Jonkman NH, Westland H, Groenwold RHH, Ågren S, Atienza F, Blue L, et al. Do self-management interventions work in patients with heart failure? an individual patient data meta-analysis. Circulation. 2016;133(12):1189–98. doi: 10.1161/CIRCULATIONAHA.115.018006 26873943 PMC5180429

[10] Kitko L, McIlvennan CK, Bidwell JT, Dionne-Odom JN, Dunlay SM, Lewis LM, et al. Family caregiving for individuals with heart failure: a scientific statement from the American heart association. Circulation. 2020;141(22):e864–78. doi: 10.1161/CIR.0000000000000768 32349542

[11] Scott Duncan T, Riggare S, Bylund A, Hägglund M, Stenfors T, Sharp L, et al. Empowered patients and informal care-givers as partners?-a survey study of healthcare professionals’ perceptions. BMC Health Serv Res. 2023;23(1):404. doi: 10.1186/s12913-023-09386-8 37101266 PMC10131407

[12] Vellone E, Biagioli V, Durante A, Buck HG, Iovino P, Tomietto M, et al. The influence of caregiver preparedness on caregiver contributions to self-care in heart failure and the mediating role of caregiver confidence. J Cardiovasc Nurs. 2020;35(3):243–52. doi: 10.1097/JCN.0000000000000632 32084078

[13] Jaarsma T, Cameron J, Riegel B, Stromberg A. Factors related to self-care in heart failure patients according to the middle-range theory of self-care of chronic illness: a literature update. Curr Heart Fail Rep. 2017;14(2):71–7. doi: 10.1007/s11897-017-0324-1 28213768 PMC5357484

[14] Grant JS, Graven LJ. Problems experienced by informal caregivers of individuals with heart failure: An integrative review. Int J Nurs Stud. 2018;80:41–66. doi: 10.1016/j.ijnurstu.2017.12.016 29353711

[15] Reinhard SC, Given B, Petlick NH, Bemis A. Advances in patient safety supporting family caregivers in providing care. In: Hughes RG, editor. Patient Safety and Quality: An Evidence-Based Handbook for Nurses. Rockville (MD): Agency for Healthcare Research and Quality (US); 2008. 21328765

[16] Harrell FE Jr, Lee KL, Califf RM, Pryor DB, Rosati RA. Regression modelling strategies for improved prognostic prediction. Stat Med. 1984;3(2):143–52. doi: 10.1002/sim.4780030207 6463451

[17] Vellone E, Riegel B, Cocchieri A, Barbaranelli C, D’Agostino F, Antonetti G, et al. Psychometric testing of the self-care of heart failure index version 6.2. Res Nurs Health. 2013;36(5):500–11. doi: 10.1002/nur.21554 23832431

[18] Graven LJ, Durante A, Abbott L, Bassi E, Howren MB, Grant JS. Self-care problems and management strategies experienced by rural patient/caregiver dyads living with heart failure: a qualitative study. J Cardiovasc Nurs. 2024;39(3):207–18. doi: 10.1097/JCN.0000000000001056 37955387

[19] Iovino EA, Koslouski JB, Chafouleas SM. Teaching simple strategies to foster emotional well-being. Front Psychol. 2021;12:772260. doi: 10.3389/fpsyg.2021.772260 34858296 PMC8631539

[20] Karimi P, Mohammadi MA, Dadkhah B, Mozaffari N. The relationship between caregiver contributions to self-care and quality of life in heart failure patients in Ardabil hospitals in Ardebil-Iran. Int J Afr Nurs Sci. 2023;18:100511. doi: 10.1016/j.ijans.2022.100511

[21] Clements L, Frazier SK, Lennie TA, Chung ML, Moser DK. Improvement in heart failure self-care and patient readmissions with caregiver education: a randomized controlled trial. West J Nurs Res. 2023;45(5):402–15. doi: 10.1177/01939459221141296 36482693

[22] Britton LE, Kaur G, Zork N, Marshall CJ, George M. “We tend to prioritise others and forget ourselves”: How women’s caregiving responsibilities can facilitate or impede diabetes self-management. Diabet Med. 2023;40(3):e15030. doi: 10.1111/dme.15030 36537593 PMC10231690

[23] Cuoco A, Younas A, Boyne J, Juarez-Vela R, M Rice B, Vellone E, et al. Perception and challenges of time management for caregivers of people with heart failure: a qualitative study. J Cardiovasc Nurs. 2024;39(6):525–34. doi: 10.1097/JCN.0000000000001027 37550836

[24] Ploeg J, Garnett A, Fraser KD, Baird LG, Kaasalainen S, McAiney C, et al. The complexity of caregiving for community-living older adults with multiple chronic conditions: A qualitative study. J Comorb. 2020;10:2235042X20981190. doi: 10.1177/2235042X20981190 33403202 PMC7739080

[25] Ajithakumari G, Hemavathy V. Caregivers burnout syndrome: support yourself while caring for a loved one. Cardiometry. 2022;(24):965–9. doi: 10.18137/cardiometry.2022.24.965969

[26] Wilson AMMM, Almeida GSM de, Santos B de CFD, Nakahara-Melo M, Conceição AP da, Cruz D de ALM da. Factors associated with caregivers’ contribution to self-care in heart failure. Rev Lat Am Enfermagem. 2022;30:e3632. doi: 10.1590/1518-8345.5838.3632 35976358 PMC9364777

[27] Johnson AM. The Effect of Caregiver Communication on Self-Care Outcomes for African Americans With Heart Failure: Walden Dissertations and Doctoral Studies; 2023.

[28] Bidwell JT, Conway C, Babicheva V, Lee CS. Person with heart failure and care partner dyads: current knowledge, challenges, and future directions: state-of-the-art review. J Card Fail. 2023;29(8):1187–206. doi: 10.1016/j.cardfail.2023.02.017 36958392 PMC10514243

[29] Nemcikova M, Katreniakova Z, Nagyova I. Social support, positive caregiving experience, and caregiver burden in informal caregivers of older adults with dementia. Front Public Health. 2023;11:1104250. doi: 10.3389/fpubh.2023.1104250 36761127 PMC9905841

[30] Stawnychy MA, Vellone E, Zeffiro V, Teitelman AM, Maria MD, Riegel B. Dyad gender and relationship quality influence heart failure self-care. Clin Nurs Res. 2023;32(1):29–39. doi: 10.1177/10547738221119338 36168717

[31] Sterling MR, Barbaranelli C, Riegel B, Stawnychy M, Ringel JB, Cho J, et al. The influence of preparedness, mutuality, and self-efficacy on home care workers’ contribution to self-care in heart failure: a structural equation modeling analysis. J Cardiovasc Nurs. 2022;37(2):146–57. doi: 10.1097/JCN.0000000000000768 33315614 PMC8196074

[32] Cohen SA, Cook SK, Sando TA, Brown MJ, Longo DR. Socioeconomic and demographic disparities in caregiving intensity and quality of life in informal caregivers: a first look at the national study of caregiving. J Gerontol Nurs. 2017;43(6):17–24. doi: 10.3928/00989134-20170224-01 28253411

[33] Bidwell JT, Vellone E, Lyons KS, D’Agostino F, Riegel B, Juárez-Vela R, et al. Determinants of heart failure self-care maintenance and management in patients and caregivers: a dyadic analysis. Res Nurs Health. 2015;38(5):392–402. doi: 10.1002/nur.21675 26355702 PMC4654948

[34] Committee on Family Caregiving for Older A, Board on Health Care S, Health, Medicine D, National Academies of Sciences E, Medicine. Families caring for an aging america. In: Schulz R, Eden J, editors. Washington (DC): National Academy of Sciences; 2016. 27905704

[35] Gary R, Dunbar SB, Higgins M, Butts B, Corwin E, Hepburn K, et al. An intervention to improve physical function and caregiver perceptions in family caregivers of persons with heart failure. J Appl Gerontol. 2020;39(2):181–91. doi: 10.1177/0733464817746757 29347863 PMC6026574

